# *Helicobacter pylori*: A Beneficial Gastric Pathogen?

**DOI:** 10.3389/fmed.2014.00026

**Published:** 2014-08-25

**Authors:** Amin Talebi Bezmin Abadi

**Affiliations:** ^1^Department of Medical Microbiology, University Medical Center Utrecht, Utrecht, Netherlands; ^2^Department of Bacteriology, Faculty of Medical Sciences, Tarbiat Modares University, Tehran, Iran

**Keywords:** *Helicobacter pylori*, pathogen, digestive diseases, human gastric mucosa, colonization, GERD

## Introduction

Since *Helicobacter pylori* (*H. pylori*) is the first successful culture three decades ago, ongoing perspectives regarding the relationship between the bacterium and human health have changed radically (1, 2). Apart from tremendous studies performed during the last years, there are still many debates regarding the unclear rationale for existence of such bacteria in human stomach (3, 4). Basically, due to the beneficiary effects of *H. pylori* colonization (regression in child asthma and other allergic disorders), it has been concluded that *H. pylori* is a common flora or at least harmless bacterium (5–10), Conversely, because of the causative role of *H. pylori* in certain digestive diseases such as duodenal ulcer and gastric cancer, other reports are quite contradictory (11–13). A large number of discussions led to a consensus regarding presence of *H. pylori* in the human stomach (5, 11, 14, 15). Our knowledge about biology of *H. pylori* suggests that the bacterium is highly adapted to stay in gastric mucosa for long time (1, 16). Indeed, living in lower surface of gastric mucosa, with no bacterial competition, provided a novel place to survive. Moreover, *H. pylori* is able to multiply freely due to the protective effects by mucosal layer. Thus, *H. pylori* had an opportunity to thrive in stomach over the course of tens of thousands of years of co-evolution with humans (17). Additionally, the high frequency of mutation in the genome also led to higher chances of survival, and natural selection helped them to remain and cause chronic infection (18). Determining whether *H. pylori* is beneficial or detrimental in human stomach has been a challenging area of research in gastroenterology (5, 11, 19, 20). In this article, we aim to elucidate various aspects of this persistent colonization of this beneficial infection.

### *H. pylori*: Carried by human over the history

It seems that *H. pylori* is an old recognized bacterium, which is not clinically comparable with new discovered infectious agents such as human immunodeficiency virus (HIV). In other words, HIV was introduced to human hosts <50 years ago. Given a long period of *H. pylori* colonization in the human stomach, mutual benefits obliged both partners to adapt themselves in order to establish stable symbiosis. It has been firmly established that *H. pylori* first subverts innate immunity and then modulates the adaptive immune system (blocking the activation of both B and T cells) (21–23). As a result, *cagA* and *vacA*, the main bacterial products, will inhibit B cell and T cell proliferation, respectively. Accordingly, immune response in the stomach is silenced against digested microbes (24, 25). Undeniably, the stomach, with its harsh acidic condition, is a container for many digested microbes every day. Possibly, regulation and modulation of immune response arose after microbial exposure to *H. pylori* colonizing the stomach (24, 25). Our current understanding of the strategies used by this pathogen to make a lifelong colonization, disclosed that maybe for our old ancestors having this bacteria in the stomach happened initially by accident, but due to natural selection, *H. pylori* made a set of adaptations, enabling the bacterium to survive and also thrive in the surface of human gastric epithelial cells. To everyone’s surprise, after millions years of living in human stomach, *H. pylori* became a strategic member of our microbiome (26). Undoubtedly, it is no exaggeration to say that both host and the bacterium are following a constant beneficial relationship, which is quite unique in biological world. After long period of *H. pylori* gastric colonization, it has evolved into a highly adaptable persistent bacterium, obtaining all necessary features of the most successful human pathogen.

### GERD and *H. pylori*: A good example of beneficial effects

Gastroesophageal reflux disease (GERD) incidence has been increased mostly in developed communities where *H. pylori* infection is almost effectively eradicated (27, 28). GERD is the main risk factor for Barrett’s esophagus, and it has been associated with another deadly gastroduodenal carcinoma called “esophageal adenocarcinoma” (3, 5). However, the relationship between GERD and *H. pylori* remains incompletely defined (29). Several studies suggested that eradication of *H. pylori* infection in the setting of duodenal ulcer disease would result in an increase in GERD symptoms (3, 30). In other words, an inverse association of *H. pylori* infection with decreased rate of this sort of disease was a challenging topic in new gastroenterology (14, 31–33). Of note, GERD and its sequelae, which include Barrett’s esophagus and esophageal adenocarcinoma, is decreasing in countries in which most individuals are infected by *H. pylori* (15, 34, 35). Actually, not only has worldwide *H. pylori* prevalence changed in recent decades but also other environmental factors affecting on human health such as socioeconomical levels, diet, and vaccination were drastically changed (10, 17). If so, *H. pylori* is confronting with different situation rather than before. Remarkably, *H. pylori* can undergo drastic genetic change through each generation, while human genes do not change frequently. As a result, frequent genetic changes in *H. pylori* helped the bacterium to adapt quickly (18). Remarkably, slower adaptation in humans deteriorates longtime established equilibrium between *H. pylori* and human. Because of this, elimination of *H. pylori* as permanent resident of human microbiome would not be the first option to deal with gastroduodenal diseases. According to what explained about GERD and *H. pylori* as a protective effect, the long cohabitation in our stomach calls for more deep studies to elucidate microbiota and human health. GERD is the best example of disease, which became more frequent after starting the *H. pylori* treatment (34). Indeed, after antibiotic usage against *H. pylori* and, of course, its eradication in Western countries, a constant equilibrium between *H. pylori* and human health disappeared. Interestingly, in Northeastern Malaysia, the low prevalence of *H. pylori* infection was frequently reported (36–38). As general rule, one expects that frequency of diseases such as asthma and GERD should be relatively low rather than the findings of expected inverse association were not found (39–41). Actually, the association between *H. pylori* infection and certain diseases such as GERD and asthma risk can be affected by geographical and genetic differences (37, 41). As a result, there is a complex and mostly undetermined associations between human microbiome and health; accordingly, all attempts to change this arranged biological system can exacerbate certain diseases. Undoubtedly, we need to eradicate virulent *H. pylori* in people with adverse clinical manifestations, but this conclusion cannot be generalized to all *H. pylori* positive subjects.

## Future of *H. pylori*

The interesting relation between *H. pylori* and humans has shown that gastric acidic condition and human immune responses, which resulted in highly adaptable microbe. Currently, half of the world population is carrying strains that can survive and multiply in human gastric mucosa. Nowadays, available data about microbiota are sharply increasing; hence, one can expect to elucidate more details about this mysterious part of our bodies. In fact, experiments, which determine an actual association between *H. pylori* and microbiota, can increase our knowledge regarding this persistent resident of our stomach. Strikingly, both approaches, (i) bacterial eradication for asymptomatic individuals and (ii) global vaccination programs do not seem necessary in current clinical setting. Alternatively, we can suggest to only eradicating *H. pylori* in patients with adverse clinical presentations. In the end, with continuing current approach against *H. pylori*, we will lose this old ancient member of our microbiota; an event, which we are not fully aware of its drawbacks.

## Conflict of Interest Statement

The author declares that the research was conducted in the absence of any commercial or financial relationships that could be construed as a potential conflict of interest.
